# Corrosion Resistance and Surface Bioactivity of Ti6Al4V Alloy after Finish Turning under Ecological Cutting Conditions

**DOI:** 10.3390/ma14226917

**Published:** 2021-11-16

**Authors:** Kamil Leksycki, Agnieszka Kaczmarek-Pawelska, Kamil Ochał, Andrzej Gradzik, Danil Yurievich Pimenov, Khaled Giasin, Daniel Chuchala, Szymon Wojciechowski

**Affiliations:** 1 Institute of Mechanical Engineering, University of Zielona Gora, 4 Prof. Z. Szafrana Street, 65-516 Zielona Gora, Poland; a.kaczmarek@iim.uz.zgora.pl; 2 Department of Materials Science, The Faculty of Mechanical Engineering and Aeronautics, Rzeszow University of Technology, Powstancow Warszawy 12, 35-959 Rzeszow, Poland; kochal@prz.edu.pl (K.O.); andrzej_gradzik@prz.edu.pl (A.G.); 3 Research and Development Laboratory for Aerospace Materials, Rzeszow University of Technology, Zwirki i Wigury 4, 35-036 Rzeszow, Poland; 4 Department of Automated Mechanical Engineering, South Ural State University, Lenin Prosp. 76, 454080 Chelyabinsk, Russia; 5 School of Mechanical and Design Engineering, University of Portsmouth, Portsmouth PO1 3DJ, UK; khaled.giasin@port.ac.uk; 6 Faculty of Mechanical Engineering and Ship Technology, Gdansk University of Technology, 11/12 Gabriela Narutowicza Street, 80-233 Gdansk, Poland; daniel.chuchala@pg.edu.pl; 7 Faculty of Mechanical Engineering, Poznan University of Technology, 60-965 Poznan, Poland

**Keywords:** Ti6Al4V alloy, finish turning, surface topography, cooling conditions, corrosion resistance, surface bioactivity, simulated body fluid (SBF), minimum quantity lubrication (MQL)

## Abstract

The influence of cooling conditions and surface topography after finish turning of Ti6Al4V titanium alloy on corrosion resistance and surface bioactivity was analyzed. The samples were machined under dry and minimum quantity lubrication (MQL) conditions to obtain different surface roughness. The surface topographies of the processed samples were assessed and measured using an optical profilometer. The produced samples were subjected to electrochemical impedance spectroscopy (EIS) and corrosion potential tests (*E*_corr_) in the presence of simulated body fluid (SBF). The surface bioactivity of the samples was assessed on the basis of images from scanning electron microscopy (SEM) and energy-dispersive spectroscopy (EDS) analysis. The inspection of the surfaces of samples after turning under dry and MQL conditions revealed unevenly distributed precipitation of hydroxyapatite compounds (Ca/P) with a molar ratio in the range of 1.73–1.97. Regardless of the cutting conditions and surface roughness, the highest values of *E*_corr_ ~0 mV were recorded on day 7 of immersion in the SBF solution. The impedance characteristics showed that, compared to the MQL conditions, surfaces machined under dry conditions were characterized by greater resistance and the presence of a passive layer on the processed surface. The main novelty of the paper is the study of the effect of ecological machining conditions, namely, dry and MQL cutting on the corrosion resistance and surface bioactivity of Ti6Al4V titanium alloy after finish turning. The obtained research results have practical significance. They can be used by engineers during the development of technological processes for medical devices made of Ti6Al4V alloy to obtain favorable functional properties of these devices.

## 1. Introduction

An important aspect that proves the usability and longevity of metals in the human body is their corrosion resistance [1]. As metal implants are surrounded by aggressive body fluids constituting the internal environment of the human body, their corrosion cannot be avoided [2]. Ti6Al4V titanium alloy is very commonly used in medical devices and applications [3,4]. In the environment of the human body, it exhibits neutral behavior and high corrosion resistance, making it a biocompatible material [5]. Titanium and its alloys belong to the group of materials with high corrosion resistance, resulting from their ability to passivate, and, in environments containing high concentrations of chloride or sulfide ions, they undergo pitting corrosion [6,7,8,9]. An important aspect of implant manufacturing is the quality of the implant surface. For orthopedic implants, it is important to achieve a surface with a designated roughness due to the need for a durable biomaterial–tissue interface. For implants and surgical instruments that are in direct contact with blood, it is important to achieve the lowest possible surface roughness. Producing surfaces with assumed roughness maintains mechanical stability and allows bone tissue to grow into and adhere to the implant surface. Higher implant surface roughness results in more favorable cell adhesion and a more accurate bone–implant fusion [10]. However, higher surface roughness contributes to the development of the implant corrosion process. This is related to the characteristic post-treatment changes present on the surface, which contribute to the formation of corrosion foci [11]. The surface roughness of titanium alloys for both medical and other industries depends on the type and conditions of the treatment it is subjected to [12]. Although titanium alloy Ti6Al4V shows very good mechanical properties and is a widely known material used in industry, it belongs to the group of materials that are difficult to process [13,14]. Due to its properties, high temperature is generated within the cutting zone during machining and adversely affects the quality of the processed surface [15,16].

Previous studies have analyzed the influence of cutting conditions and surface roughness on the corrosion resistance of medical-grade materials in various solutions. Bertolini et al. [17] investigated the corrosion resistance of Ti6Al4V alloy after turning under dry and cryogenic conditions. Cutting speeds (*v_c_*) of 50 and 80 m/min, feed rates (*f*) of 0.1 and 0.2 mm/rev, and a depth of cut (*a_p_*) of 0.25 mm were applied. The corrosion tests were performed in 0.9% NaCl solution. It was shown that cryogenic processing effectively improves the corrosion and fretting properties of the examined material. Bertolini et al. [18] studied the effect of feed rate and dry and cryogenic conditions on the corrosion behavior of AZ31 alloy. A feed rate within the 0.01–0.1 mm/rev range, cutting speed of 100 m/min, and depth of cut of 0.25 mm were applied. The tests were performed in simulated body fluid (SBF). The feed rate affected the surface roughness and corrosion behavior of AZ31 alloy. The corrosion resistance of AZ31 magnesium alloy was more favorable using a lower feed rate and cryogenic conditions. Pu et al. [19] investigated the corrosion resistance of magnesium alloy AZ31B-O following dry and cryogenic cutting conditions. A tool edge radius of 30 and 70 μm, feed rate of 0.1 mm/rev, and cutting speed of 100 m/min were used. The samples were tested in SBF and 5% NaCl solution. Compared to dry machining, cryogenic ensure better surface integrity and corrosion resistance. Bruschi et al. [20] investigated the corrosion behavior of 316L stainless steel to increase its resistance to sterilization cycles. The processing was carried out under low-temperature conditions, and the results were compared with the dry and wet conditions. The following parameters were applied: *v_c_* = 200 m/min, *f* = 0.2 mm/rev, and *a_p_* = 0.3 mm. The tests were performed in a commercial IGENAL-N sterilization solution. Corrosion resistance improved with low-temperature conditions and deteriorated after dry conditions. Bertolini et al. [21] analyzed the corrosion behavior of stainless steel 316L following low-temperature and wet conditions. The following parameters were applied: *v_c_* = 200 m/min, *f* = 0.2 mm/rev, and *a_p_* = 0.3 mm. The tests were performed in a commercial IGENAL-N sterilization solution. Low-temperature conditions ensure a hardened and more elastic surface layer, which, compared to wet conditions, increases the general and local corrosion resistance of the tested steel.

Zhang and Liu [22] evaluated the surface topography of Ti6Al4V alloy after finish turning under dry conditions. The values of *R_a_* and *R_z_* parameters increased, and the shapes of surface roughness profiles changed with increasing feed rate. Sartori et al. [23] investigated the surface integrity of Ti6Al4V alloy after cryogenic and dry cutting conditions. Surface topography deteriorated with cryogenic conditions, but fewer surface defects were observed. Mia et al. [24] investigated the surface roughness of Ti6Al4V alloy after turning under cryogenic liquid nitrogen (LN2) conditions. LN2 dual jets improve the surface quality. The following cutting parameters were recommended: *v_c_* = 140 m/min and *f* = 0.16 mm/rev. Deiab et al. [25] assessed the effect of dry, cooled air, flood, cryogenic, minimum quantity lubrication (MQL), and minimum quantity cooled lubrication (MQCL) conditions on the surface roughness of Ti6Al4V alloy. At a higher machining speed and feed rate in dry and MQL conditions, the lowest *R_a_* parameter values were obtained. Sun et al. [26] analyzed the surface roughness of Ti-5553 alloy under cryogenic conditions, and the results were compared to wet and MQL conditions. The smallest values of the *R_a_* parameter were obtained from MQL. Sun et al. [27] investigated the surface integrity after turning titanium alloy TB6 under dry conditions. A decrease in the *R_a_* parameter value was observed with decreasing feed rate. Fewer defects on the surface were observed with increasing cutting speed. Gupta et al. [28] analyzed the effect of Ranque-Hilsch vortex fluid-assisted minimum quantity cutting fluids (RHVT-MQCF) and MQL cutting conditions on surface roughness after turning pure titanium. Favorable results were obtained for the machining conditions with RHVT-MQCF.

There are few studies in the open literature which looked into the effect of surface topography and cutting conditions on the corrosion resistance after finishing turning of titanium alloys. Yet, the processing conditions affect both the surface topography [29] and the corrosion resistance [30]. The existing articles do not consider the influence of modern cooling conditions, i.e., near-dry cutting, which improves the surface quality [31] while offering numerous economic and environmental benefits [32]. In addition, there is no information on the effect of surface topography and cooling conditions on surface bioactivity after the turning process of titanium alloys, which can be considered a novelty in this research work. As studies have shown, surface topography affects surface bioactivity. Ravelingien et al. [33] investigated the effect of surface topography on the surface bioactivity of Ti6Al4V alloy after alkali treatment. The titanium plates had different surface roughness (*R_a_* = 0.13 μm, 0.56 μm, 0.83 μm, and 3.63 μm) and were prepared by Al_2_O_3_ grit-blasting. For samples with *R_a_* = 0.56 μm, complete hydroxyapatite (HA) coating was obtained after 7 days of storage in SBF. Constantinescu et al. [34] studied the effect of surface roughness of Ti6Al4V alloy on the shape, size, and distribution of HA. The samples were sandblasted with Al_2_O_3_ particles of different sizes. It was found that surface roughness affects the morphology and distribution of HA. Demirci et al. [35] analyzed the effect of surface modifications of Ti6Al4V alloy on the ability to form apatite. Samples were produced using the additive method (AM) using varying laser powers. The samples were stored in SBF for 2 weeks. Depending on the production laser power, surface roughness and topography, as well as wettability and microstructure, affected the formation of apatite on the surface.

A high surface bioactivity of titanium alloys, shaped by various techniques to produce orthopedic implants, is achieved by anodizing the surface in solutions containing phosphate ions or by forming layers with morphology favoring the surface bioactivity [36]. Increasing the surface bioactivity of titanium implants can also be achieved by covering their surface with a layer containing calcium and phosphorus hydroxyapatite. HA-coated titanium alloys exhibit higher surface bioactivity than uncoated ones [37]. HA coating aids implant fixation and increases implant life. The dense layer of HA coating on the implant surface has a beneficial effect on biointegration, improves the stability of the implant and bone tissue junction, and minimizes the release of metal ions in the human body [38]. Hydroxyapatite is a ceramic material that is a natural bone component. In the assessment of the in vitro surface bioactivity of titanium alloys, it was verified, inter alia, whether hydroxyapatite is released on a given surface of the alloy following processing [39]. HA is like a bioceramic that has very good biological properties facilitating bone repair and reconstruction [40]. The surface roughness of titanium alloys suitable for the growth of osteoblasts is 0.2–0.7 μm, and, in the literature, many reports can be found on the protective and bioactive layers on titanium alloys and their corrosion resistance [41,42,43,44].

Summarizing the literature review, it was found that, in the research works to date, there is a lack of information on the effect of finish turning of Ti6Al4V titanium alloy under ecological conditions on the corrosion resistance and surface bioactivity in SBF solution, whose chemical composition is most similar to human blood plasma. In the research, the simultaneous effect of feed rate and surface roughness on these performance properties of Ti6Al4V titanium alloy has also basically not been analyzed. This can be considered a research gap. Therefore, the current study aimed to analyze the effect of dry and MQL conditions and surface topography after finish turning of Ti6Al4V titanium alloy on corrosion resistance and surface bioactivity in SBF solution. This research is of practical importance and can be used as a step in the development of technological processes in the production of medical devices, to ensure favorable operating properties of these devices.

## 2. Materials and Methods

### 2.1. Workpiece Details and Preparation

Ti6Al4V alloy, with the chemical composition and mechanical properties compliant with ISO 5832-3:2016 [45] standard, was examined (Table 1). Ti6Al4V alloy is characterized by high strength-to-weight ratios and has high corrosion resistance; thus, it is used in many industries including medical, automotive, aerospace, and marine [46]. 

The machining was performed on a CNC lathe CKE6136i (Dalian Machine Tool Group Corporation, Dalian, China) using a turning with a CoroTurn SDJCR 2020K 11 (Sandvik Coromant, Sandviken, Sweden) tool holder and a CoroTurn DCMX 11 T3 04-WM 1115 (Sandvik Coromant, Sandviken, Sweden) insert. The geometry of the cutting insert was as follows: *κ*_r_ = 93°, γ_o_ = 18°, α_o_ = 7°, *r*_ε_ = 0.4 mm. The material used for the cutting insert was cemented carbide GC 1115 with (Ti, Al)N + (Al, Cr)_2_O_3_ coating deposited by Physical Vapour Deposition (PVD). The inserts were changed after each test, so each sample was machined with a new and sharp edge. The samples were machined under dry and MQL conditions because today’s manufacturing industry is very much focused on environmental protection [47] and a reduction in the consumption of machining fluids [48]. Furthermore, MQL has been successfully applied to improve the machinability of difficult-to-cut materials [49,50]. In the MQL method, we used ECOCUT MIKRO 20 E oil, which was produced by mixing with air. The oil mist was produced using the Lenox 1LN Mikronizer (Lenox, East Longmeadow, MA, USA). The preferred oil mist formation conditions suggested by Maruda et al. were applied [51]: oil flow 39.4 mL/h, airflow 5.8 L/m, pressure 0.48 MPa, and 0.2 m distance of the nozzle from the point of contact between the material and the cutting edge. The samples with minimum (*R_a_*_min_ = 0.29–0.37 μm) and maximum (*R_a_*_max_ = 1.62–2.22 μm) surface roughness obtained after finish turning were examined. Surface roughness affects the corrosion of metals and, as commonly known, depends on the feed rate (*f*). For samples with *R_a_*_min_, a lower feed rate was used for MQL conditions (*f* = 0.1 mm/rev) to obtain surface roughness parameters similar to dry machining (*f* = 0.14 mm/rev). Regardless of the machining conditions, the feed rate for the samples with *R_a_*_max_ was 0.35 mm/rev. Due to the small diameter of the sample, a constant cutting speed (*v_c_*) of 80 m/min was set, as well as a constant cutting depth (*a_p_*) of 0.5 mm, typical for the finish cutting. Both the length and the diameter of the samples were 15 mm.

### 2.2. Measurement of Surface Roughness Metrics

The processed surface was examined using a Sensofar S Neox 3D optical profilometer (Sensofar Group, Barcelona, Spain), and the measurement results were analyzed using Mountains Maps Premium 7.4 software (Digital Surf, Besançon, France). The measurement area used was 1.30 × 1.75 mm. Selected parameters of the surface roughness amplitude were analyzed in accordance with the ISO 4287:1999 [52] standard *Ra* (arithmetic mean deviation of the roughness profile), *R_z_* (maximum height of the roughness profile), *R_p_* (maximum peak height of the roughness profile), *R_v_* (maximum valley depth of the roughness profile), *R_sk_* (skewness of the roughness profile), *R_ku_* (kurtosis of the roughness profile)*,* roughness profiles, and 2D topographies of the processed surface. In industry, parameters *R_a_* and *R_z_* are widely used to evaluate product quality. The parameters *R_sk_* and *R_ku_* can be complementary to the parameters *R_a_* and *R_z_* and allow the evaluation of the functional properties of the surface [53,54]. The tests were repeated three times and the standard deviation did not exceed 5%.

Before corrosion resistance and surface bioactivity testing, the samples were washed in an IS-1 (Intersonic, Olsztyn, Poland) ultrasonic washer in distilled water to remove machining residues, such as machined material or oil that sticks to the surface. Samples of Ti6Al4V titanium alloy were subjected to electrochemical tests using the Atlas 0531 Electrochemical Unit and Impedance Analyzer potentiostat/galvanostat (Atlas-Sollich, Rebiechowo, Poland). The chemical composition of the simulated body fluid can be found in [55]. The SBF used in this study had a temperature of 37 ± 1 °C, and a pH in the range of 7.2–7.4. An area of 0.25 cm^2^ was marked on each sample, which was then exposed to the electrolyte. The remainder of the sample was covered with a layer of paint. The tests were repeated three times and the standard deviation did not exceed 3%.

The samples were subjected to electrochemical impedance spectroscopy (EIS) tests, preceded by a 1 min measurement of the corrosion potential (*E*_corr_). EIS studies were performed according to ISO 16773-2:2016 [56] and ISO 16773-3:2016 [57] standards. The tests were carried out in a three-electrode system, in which the reference electrode was silver chloride, with a platinum plate as the counter electrode. Changes in electrochemical properties of the samples were recorded after 1 h and then after 1, 7, 14, 28, 46, and 72 days of immersion in SBF solution. EIS spectra were recorded within the 10^−5^–0.1 Hz frequency range. The results obtained were analyzed, and Bode and Nyquist charts were plotted. The sample surfaces were assessed on the basis of SEM images and EDS analysis obtained using a JSM-5600LV scanning microscope (JEOL, Tokyo, Japan) with an EDS 2000 X-ray microanalyzer and an AVT-HORN camera (AVT, Aachen, Germany). To neutralize the influence of Ti, Al, and V on the effectiveness of registration of the presence of hydroxyapatite compounds on the surfaces of the tested titanium alloy samples, analyzed by EDS, these elements were excluded from the list of analyzed elements. A similar approach was put forward by Feldshtein et al. [58].

**Figure 1:**
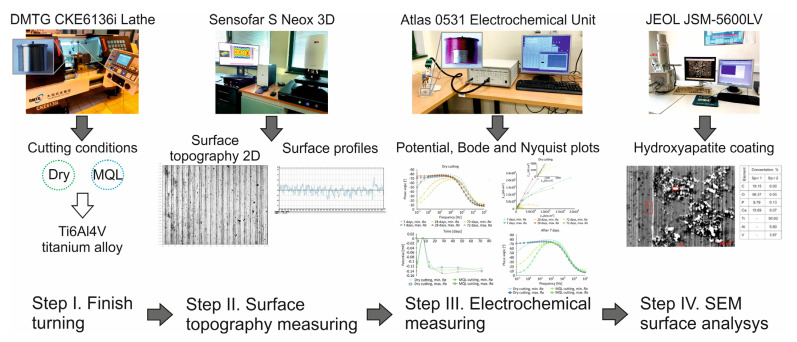
Experimental research plan.

The experimental research plan is shown in Figure 1.

## 3. Results and Discussion

Surface roughness parameters were obtained for samples with minimum and maximum *R_a_* for Ti6Al4V alloy after finish turning under the ecological cutting conditions shown in Table 2.

Garcia-Martinez et al. [59] showed that machining under MQL conditions reduces surface roughness and improves surface integrity compared to dry machining. For samples with *R_a_*_min_ after dry turning, a reduction in surface roughness parameters *R_a_*, *R_z_, R_p_, R_v_,* and *R_sk_* in the range of 6% to 165% was obtained compared to MQL conditions. On the other hand, for samples with *R_a_*_max_ machined under MQL conditions, a reduction in *R_a_*, *R_z_*, *R_p_*, and *R_v_* parameters was obtained in the range of 25% to 27%.

**Figure 2:**
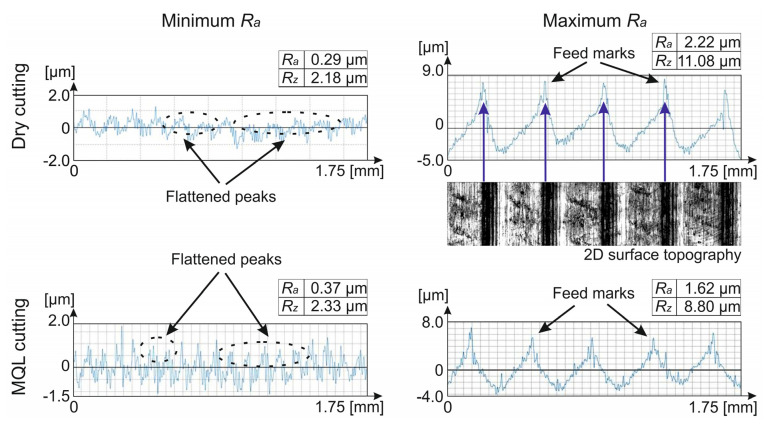
Surface roughness profiles of Ti6Al4V alloy after finish turning.

Figure 2 shows the surface roughness profiles of samples with *R_a_*_min_ and *R_a_*_max_ parameters obtained after finish turning under dry and MQL conditions.

Within the range of minimum parameter values, *R_a_* = 0.29–0.37 μm and *R_z_* = 2.18–2.33 μm, regardless of the cooling conditions, flattened peaks were observed on the surface roughness profiles attesting to the plastic deformation of the surface layers characteristic of the processed implant surface [60]. However, within the range of maximum *R_a_* = 1.62–2.22 μm and *R_z_* = 8.80–11.08 μm parameter values, there were visible feed marks. For example, Figure 2 shows a cutout of the 2D surface topography along with the surface roughness profile for a *R_amax_* sample after dry cutting.

**Figure 3:**
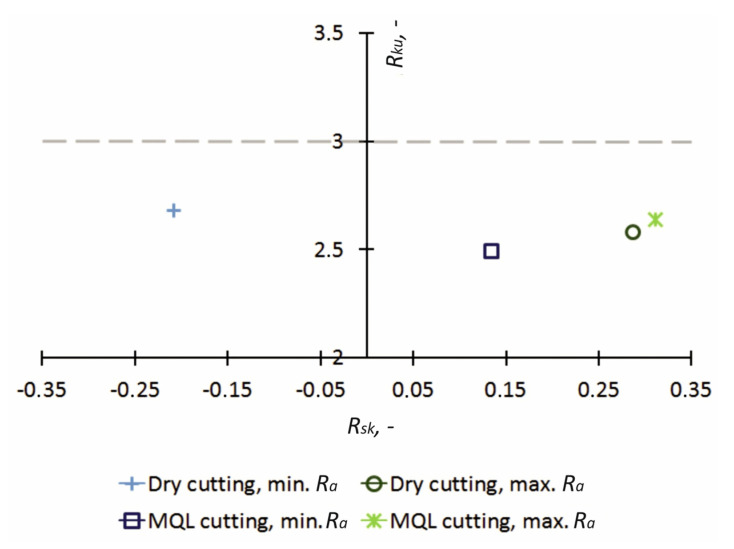
*R_ku_–R_sk_* topological map for Ti6Al4V alloy after finish turning.

The correlation of the *R_sk_* and *R_ku_* parameters yields an *R_sk_–R_ku_* topological map, which enables an evaluation of the surface irregularity distribution. The parameters *R_sk_* and *R_ku_* are responsive to high peaks and deep pits located on the machined surface [61]. The *R_sk_–R_ku_* topological map for Ti6Al4V alloy after finish turning is shown in Figure 3. Compared to the surface area of samples with *R_a_*_min_, an increase in *R_sk_* parameters was obtained for samples with *R_a_*_max_. Very high peaks and very deep pits were seen on surfaces with *R_a_*_max_, whereas high peaks and deep pits were seen on surfaces with *R_a_*_min_ machined under MQL conditions. In turn, flattened peaks were seen on the surface with *R_a_*_min_ under dry machined conditions. Regardless of the cooling conditions for the samples with *R_a_*_min_ and *R_a_*_max_, the surfaces were characterized by regular shapes, as evidenced by the parameter value *R_ku_* < 3.

**Figure 4:**
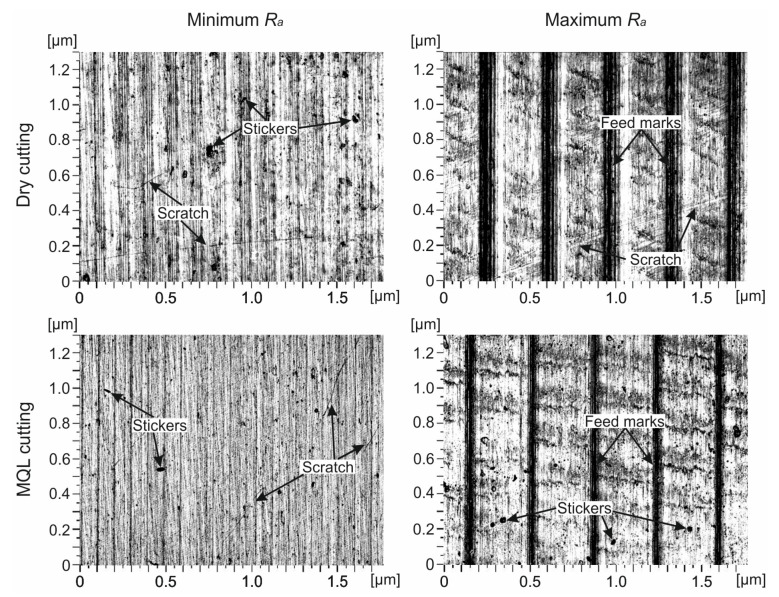
The 2D topographies of Ti6Al4V titanium alloy surface after finish turning.

Figure 4 shows 2D surface images of the samples with *R_a_*_min_ and *R_a_*_max_ after finishing turning. For *R_a_*_min_, on the surface processed under dry and MQL conditions, scratches were observed, which were caused by the unfavorable shape of the chip entangling the workpiece [62,63] and irregularly spaced stickers, resulting from the adhesive bonds of the chips to the processed surface [64]. For *R_a_*_max_, on the surface processed in the tested cooling conditions, clear traces of feed rate, which are typical of turning, were found. In addition, scratches occurred under dry cutting conditions and stickers with MQL. A summary of the surface topography results of samples with *R_a_*_min_ and *R_a_*_max_ of Ti6Al4V alloy after turning under dry and MQL conditions is presented in Table 3.

**Figure 5:**
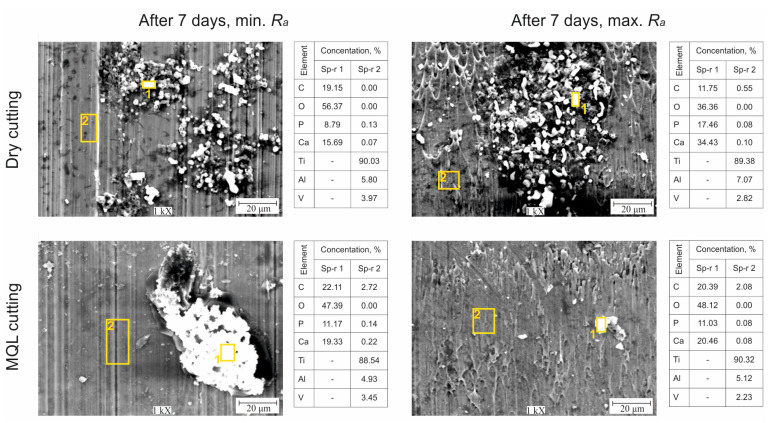
SEM pictures and EDS analysis results for Ti6Al4V alloy with *R*_amin_ and *R_a_*_max_ under dry and MQL conditions after 7 days of storage in SBF.

The results of the impedance tests showed that the cutting conditions and surface topography after finish turning of Ti6Al4V alloy affected the bioactivity of the surface and the release of hydroxyapatite and changed the electrochemical characteristics of the layer. SEM images and EDS analyses of the surfaces after finish turning after dry and MQL conditions after 7 days of storage in SBF solution are shown in Figure 5.

**Figure 6:**
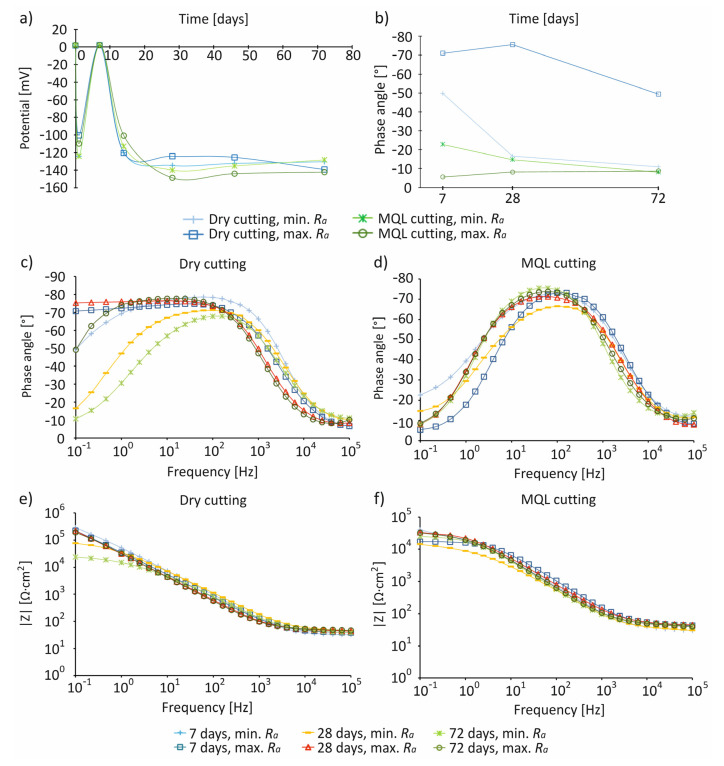
The results of electrochemical tests of Ti6Al4V alloy samples after turning under dry and MQL conditions with *R_a_*_min_ and *R_a_*_max_ stored in SBF: changes in corrosion potential for 72 days (**a**) the value of the phase angle at the frequency of 0.1 Hz after 7 days (**b**) the Bode phase plots after 7, 28, and 72 days (**c**,**d**) and the Bode plots after 7, 28, and 72 days (**e**,**f**).

Spherical precipitates unevenly covering the surface of the samples were observed for samples after turning under dry and MQL conditions. Analysis of the content of calcium and phosphorus confirmed the presence of hydroxyapatite, in which the Ca/P molar ratio was 1.73–1.78 for samples with *R_a_*_min_ and 1.85–1.97 for samples with *R_a_*_max_. It should be emphasized that the secretions of hydroxyapatite were more irregular on samples after turning with MQL conditions than on samples subjected to dry machining; furthermore, their stoichiometric composition was closest to the composition of hydroxyapatite, which is a bone component (Ca/P = 1.67) [36]. Changes in the corrosion potential of samples made of Ti6Al4V alloy with *R_a_*_min_ and *R_a_*_max_ after turning under dry and MQL conditions stored in SBF solution are shown in Figure 6a.

For samples with *R_a_*_min_ and *R_a_*_max_ after turning under dry and MQL conditions, over time after immersion in SBF, the values of the corrosion potentials decreased and then significantly increased, to reach the values of *E*_corr_ ~0 mV. Then, they decreased to *E*_corr_ ~−120 to −140 mV, which continued until the 72nd day. The increase in the value of corrosion potentials on the seventh day from the moment of immersion in SBF proves the existence of positive ion adsorption processes from the solution, forming a double electrical layer at the alloy surface, from which hydroxyapatite is released [65]. The results obtained in the first hours/days after immersion in the solution are very important, as they may indicate the body’s reaction to the metal placed in it as an implant [66].

**Figure 7:**
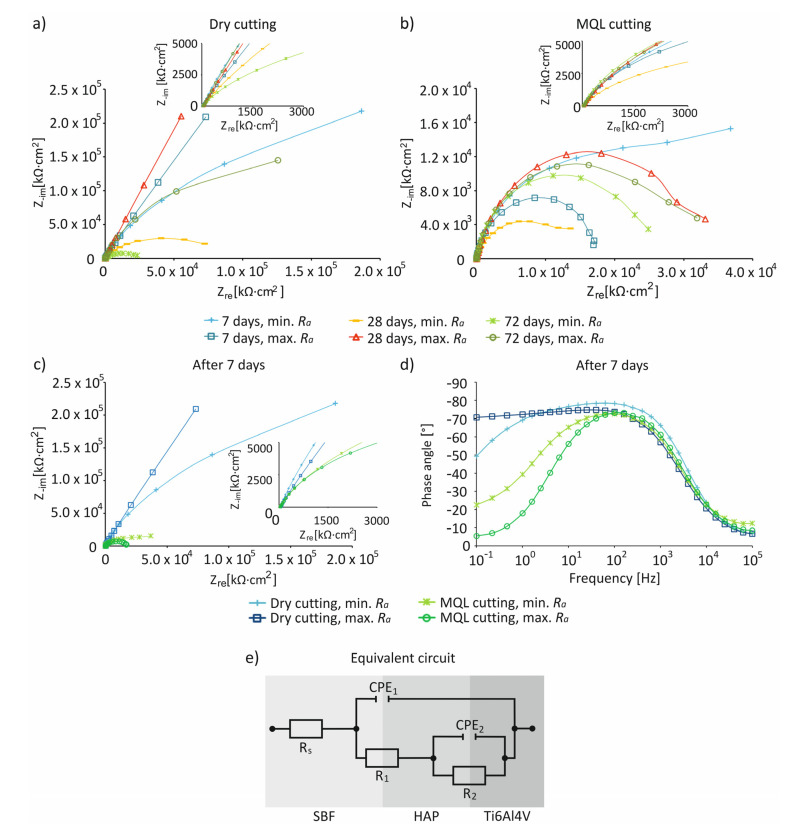
Niquist charts for samples of Ti6Al4V alloy after turning with *R_a_*_min_ and *R_a_*_max_ after 7, 28, and 72 days of storage in SBF, under dry (**a**) and MQL (**b**) conditions; comparison of impedance characteristics in Niquist representation (**c**) and Bode phase plots (**d**) with equivalent circuit illustrating the nature of samples (**e**).

The results of impedance tests for samples made of Ti6Al4V alloy with *R_a_*_min_ and *R_a_*_max_ after turning with dry and MQL conditions after 7 days of storage in SBF solution are presented in the form of Bode and Niquist diagrams in Figure 6 and Figure 7. The impedance characteristics (Figure 6 and Figure 7) indicate that, regardless of the surface roughness of samples, compared to MQL, under dry conditions, surfaces were characterized by a greater resistance and an almost capacitive response, illustrated by a phase angle close to −80° recorded in a wide frequency range by control systems including in the range of 10^−1^–10^3^ Hz, indicating the presence of a passive layer on the processed surface. The test results for the anodized titanium alloy Ti4Al4V reported in the literature show a similar characteristic [12,37,39]. In the Niquist illustration, samples after turning under MQL conditions were characterized by one loop (Figure 7b); when compared to samples with *R_a_*_max_, higher resistance values were recorded for samples with *R_a_*_min_. It is reported in the literature that, in the case of polished samples, larger diameters of the loops are observed, which are evidence of a higher resistance [12]. For samples with *R_a_*_max_, the lowest resistance occurred 7 days after immersion in SBF solution. Then, after 28 days, the resistance value increased and remained at a similar level for 72 days, suggesting that no hydroxyapatite was recorded on the surfaces of the samples during this period. The obtained data were then filtered. The equivalent circuit Rs (P1, R1(P2, R2)) provided the best fit to the test data, where Rs is the electrolyte resistance, R1 and CPE1 are the hydroxyapatite layer resistivity and metal capacity, and R2 and CPE2 are the charge transfer resistance and double-layer capacity (Figure 7e). A summary of the corrosion resistance and surface bioactivity test results of samples with *R_a_*_min_ and *R_a_*_max_ of Ti6Al4V alloy after turning under dry and MQL conditions is presented in Table 4.

## 4. Conclusions

The aim and novelty of this work was to analyze the effect of ecological dry and MQL machining conditions and surface topography of Ti6Al4V titanium alloy after finish turning on corrosion resistance and surface bioactivity. The surface topographies of the processed samples were assessed and measured using a Sensofar S Neox 3D optical profilometer. Samples with different surface roughness were subjected to electrochemical impedance spectroscopy and corrosion potential tests in the presence of simulated body fluid. The following was established:

1. For samples with *R_a_*_min_ after dry turning, a reduction in surface roughness parameters of 6% to 165% was obtained compared to MQL conditions. On the other hand, for samples with *R_a_*_max_ machined under MQL conditions, a reduction was obtained of 25% to 27%.
2. Regardless of the cutting conditions within the minimum range *R_a_* = 0.25–0.37 μm, flattened peaks were observed on the surface roughness profiles, and, within the range of the maximum *R_a_* = 1.62–2.22 μm, there were visible feed marks.
3. *R_sk_–R_ku_* topological map showed very high peaks and very deep pits on surfaces with *R_a_*_max_, as well as high peaks and deep pits on surfaces with *R_a_*_min_, machined under MQL conditions. Flattened peaks were seen on the surface with *R_a_*_max_ under dry machined conditions. On the 2D images of the surface with *R_a_*_max_, clear traces of the feed rate were recorded, and, on the surfaces with *R_a_*_min_, scratches and irregularly spaced stickers were observed under the analyzed cutting conditions.
4. After turning under dry and MQL conditions, unevenly distributed precipitates of hydroxyapatite compounds were present on the surfaces of the samples. The Ca/P molar ratio for samples with *R_a_*_min_ was within the range 1.73–1.78, whereas that for samples with *R_a_*_max_ was within the range 1.85–1.97.
5. For the studied cutting conditions and surface roughness, the highest values of *E*_corr_ ~0 mV were recorded on day 7 of immersion in the SBF solution.
6. The impedance characteristics indicated that, compared to MQL conditions, under dry conditions, surfaces were characterized by a greater resistance and an almost capacitive response, illustrated by a phase angle close to −80° recorded in a wide frequency range by control systems including in the range of 10^−1^–10^3^ Hz, indicating the presence of a passive layer on the processed surface.
7. The obtained research results have practical significance. They can be used by engineers during the development of technological processes for medical devices made of Ti6Al4V alloy, to obtain favorable functional properties of these devices, i.e., corrosion resistance and bioactivity of the surface after finish turning. Therefore, a lower surface roughness under dry conditions is recommended to achieve this success.

## Figures and Tables

**Table 1:** Chemical composition and mechanical properties of Ti6Al4V titanium alloy, According to ISO 5832-3:2016.

Chemical Composition (%)							
O	V	Al	Fe	H	C	N	Ti
<0.20	3.5	5.5	<0.30	<0.0015	<0.08	<0.05	rest
**Mechanical Properties**							
**Modulus of Elastic (MPa)**		**Tensile Strength (MPa)**		**Yield Strength (MPa)**		**Fatigue Strength (MPa)**	
110–114		960–970		850–900		620–725	

**Table 2:** Surface Roughness Parameters for Samples with *R_amin_* and *R_amax_* Obtained after Finish Turning.

Cutting Conditions	Surface Roughness Range	*R_a_* (μm)	*R_z_* (μm)	*R_p_* (μm)	*R_v_* (μm)	*R_sk_*	*R_ku_*
Dry	*R_amin_*	0.29	2.18	1.16	1.01	−0.208	2.68
MQL		0.37	2.33	1.27	1.07	0.135	2.49
Dry	*R_amax_*	2.22	11.8	7.23	4.62	0.287	2.58
MQL		1.62	8.80	5.43	3.37	0.311	2.64

**Table 3:** Summary of Surface Topography Results of Samples with *R_amin_* and *R_amax_* of Ti6Al4V Alloy after Finish Turning under Ecological Cutting Conditions.

Cutting Conditions	Surface Roughness Range	Surface Roughness Profiles	Surface Roughness Parameters	*R_ku_–R_sk_* Topological Maps	2D Surface Images
Dry	*R_amin_*	Flattened peaks	Dry compared to MQL: decrease *R_a_, R_z_, R_p_, R_v_,* and *Rsk* of 6–165%	Flattened peaks, regular shapes	Scratch, stickers
MQL				High peaks and deep pits, regular shapes	
Dry	*R_amax_*	Feed marks	MQL compared to dry: decrease *R_a_, R_z_, R_p_,* and *R_v_* of 25–27%	Very high peaks and very deep pits, regular shapes	Feed marks, scratch
MQL					Feed marks, stickers

**Table 4:** Summary of Corrosion Resistance and Surface Bioactivity Results of Samples with *R_a_*_min_ and *R_a_*_max_ of Ti6Al4V Alloy after Turning under Ecological Cutting Conditions.

Cutting Conditions	Surface Roughness Range	Precipitation of Hydroxyapatite, after 7 Days	Stoichiometric Composition (Ca/P), after 7 Days	Highest Values of *E*_corr_	Impedance Characteristics
Dry	*R_a_*_min_	Irregular, spherical	1.78	~0 mV, after 7 days	Higher resistance, an almost capacitive response, presence of a passive layer
MQL		More irregular, spherical	1.73		Low resistance, lack of presence of a passive layer
Dry	*R_a_*_max_	Irregular, spherical	1.97		High resistance, an almost capacitive response, presence of a passive layer
MQL		More irregular, spherical	1.85		Low resistance, lack of presence of a passive layer

## Nomenclature

*a_p_*	Depth of cut (mm)
*E*_corr_	Corrosion potential (mV)
EDS	Energy dispersive spectroscopy
EIS	Electrochemical impedance spectroscopy
*f*	Feed rate (mm/rev)
HA	Hydroxyapatite
MQL	Minimum quantity lubrication
*R_a_*	Arithmetic mean deviation of the roughness profile (μm)
*R_amin_*	The minimum value of the surface roughness parameter *R_a_* (μm)
*R_amax_*	The maximum value of the surface roughness parameter *R_a_* (μm)
*R_ku_*	Kurtosis of the roughness profile
*R_p_*	Maximum peak height of the roughness profile (μm)
*R_sk_*	Skewness of the roughness profile
*R_v_*	Maximum valley depth of the roughness profile (μm)
*R_z_*	Maximum height of the roughness profile (μm)
SBF	Simulated body fluid
SEM	Scanning electron microscope
*v_c_*	Cutting speed (m/min)
